# Supplementary material for: Exploiting collider bias to apply two-sample summary data Mendelian randomization methods to one-sample individual level data
Source: PLoS Genet. 2021 Aug 9;17(8):e1009703. doi: 10.1371/journal.pgen.1009703 (PMC8376220; doi:10.1371/journal.pgen.1009703)
Supplement: S1 Text — A: A formal proof of the Collider-Correction formulae. B: A list of outlying SNPs detected in analysis (a) and analysis (b) of the data example. (PDF) [file pgen.1009703.s001.pdf]

Supporting information 1A and 1B for 'Exploiting collider bias to apply two-sample summary data Mendelian randomization methods to one-sample individual level data'

July 14, 2021

A: Derivation of the Collider-Correction formula

The asymptotic least squares estimates of the effects of X and G on Y , without conditioning on U , are

$$\begin{bmatrix} \beta^* \\ \alpha_1^* \\ \vdots \\ \alpha_k^* \end{bmatrix} = \begin{bmatrix} var\left(X\right) & cov\left(X, G_1\right) & \cdots & cov\left(X, G_k\right) \\ cov\left(X, G_1\right) & var\left(G_1\right) & \cdots & cov\left(G_1, G_k\right) \\ \vdots & \vdots & \ddots & \vdots \\ cov\left(X, G_k\right) & cov\left(G_1, G_k\right) & \cdots & var\left(G_k\right) \end{bmatrix}^{-1} \begin{bmatrix} cov\left(X, Y\right) \\ cov\left(G_1, Y\right) \\ \vdots \\ cov\left(G_k, Y\right) \end{bmatrix}$$

Assuming no LD between SNPs, so $cov\left(G_i, G_j\right) = 0$ where $i \neq j$, the variance-covariance matrix has block form with a diagonal matrix in the lower right quadrant. Block-wise inversion gives

$$\begin{bmatrix} var\left(X\right) & cov\left(X, G_1\right) & \cdots & cov\left(X, G_k\right) \\ cov\left(X, G_1\right) & var\left(G_1\right) & \cdots & cov\left(G_1, G_k\right) \\ \vdots & \vdots & \ddots & \vdots \\ cov\left(X, G_k\right) & cov\left(G_1, G_k\right) & \cdots & var\left(G_k\right) \end{bmatrix}^{-1} =$$

$$\frac{1}{var\left(X\right) - \sum_j \frac{cov(X,G_j)^2}{var(G_j)}} \begin{bmatrix} 1 & \frac{-cov(X,G_1)}{var(G_1)} & \cdots & \frac{-cov(X,G_k)}{var(G_k)} \\ \frac{-cov(X,G_1)}{var(G_1)} & \frac{cov(X,G_1)^2}{var(G_1)^2} & \cdots & \frac{cov(X,G_1)cov(X,G_k)}{var(G_1)var(G_k)} \\ \vdots & \vdots & \ddots & \vdots \\ \frac{-cov(X,G_k)}{var(G_k)} & \frac{cov(X,G_1)cov(X,G_k)}{var(G_1)var(G_k)} & \cdots & \frac{cov(X,G_k)^2}{var(G_k)^2} \end{bmatrix} +$$

$$\begin{bmatrix} 0 & 0 & \cdots & 0 \\ 0 & \frac{1}{var(G_1)} & \cdots & 0 \\ \vdots & \vdots & \ddots & \vdots \\ 0 & 0 & \cdots & \frac{1}{var(G_k)} \end{bmatrix}$$

Then

$$\beta^* = \frac{cov\left(X, Y\right) - \sum_j \frac{cov(X,G_j)cov(G_j,Y)}{var(G_j)}}{var\left(X\right) - \sum_j \frac{cov(X,G_j)^2}{var(G_j)}}$$

And

$$\alpha_i^* = \frac{\frac{-cov(X,G_j)}{var(G_j)} \left(cov\left(X, Y\right) - \sum_j \frac{cov(X,G_j)cov(G_j,Y)}{var(G_j)}\right)}{var\left(X\right) - \sum_j \frac{cov(X,G_j)^2}{var(G_j)}} + \frac{cov\left(G_i, Y\right)}{var\left(G_i\right)}$$

$$= -\beta_{XG_j}\beta^* + \frac{cov\left(G_i, Y\right)}{var\left(G_i\right)}$$

From equation 2, $cov\left(G_i, Y\right) = \left(\alpha_i + \beta\beta_{XG_i}\right) var\left(G_i\right)$. Therefore

$$\alpha_j^* = \alpha_j + \beta_{XG_j}\left(\beta - \beta^*\right)$$

The causal effect β is therefore the observational effect β^* , plus the slope of the regression of α_j^* on β_{XG_j} .

B: Outlier SNPs sets for the Insomnia-HBA1c analysis.

SNP set detected as outliers using a Bonferroni corrected exact Q statistic in analysis (a) (23andMe + UK Biobank data)

1	rs10758593
2	rs1264419
3	rs12917449
4	rs12924275
5	rs1861412
6	rs214934
7	rs2737240
8	rs2792990
9	rs3131638
10	rs34490907
11	rs429358
12	rs4788203
13	rs6888135

SNP set detected as outliers using a Bonferroni corrected exact Q statistic in analysis (b) (23andMe data only)

1	rs10758593
2	rs1264419
3	rs214934
4	rs2792990
5	rs4788203
6	rs647905
