# Supplementary material for: Exploiting collider bias to apply two-sample summary data Mendelian randomization methods to one-sample individual level data
Source: PLoS Genet. 2021 Aug 9;17(8):e1009703. doi: 10.1371/journal.pgen.1009703 (PMC8376220; doi:10.1371/journal.pgen.1009703)
Supplement: Attachment — Submitted filename: review.docx [file pgen.1009703.s004.docx]

This is a quite interesting work and I greatly appreciated the very nice review of the state-of-the-art in the background section. Altogether, the authors proposed a fairly intuitive solution to improve the MR framework, estimating the average collider bias across multiple weak instruments to correct the estimator of the causal effect of the exposure on the outcome. I am actually surprised this solution has not been proposed before. The manuscript is quite dense, and my comments are mostly about providing the reader more details about the methods.

1. When describing the standard IVW method, the authors describe a two-step approach using an inverse variance meta-analysis of the ratio BetaYG over BetaXG (background, Fig 2, and Methods). However, in the attached R code, it appears they are in practice using a weighted linear regression with intercept forced at 0 (e.g. summary(lm(BetaYG~-1+BetaXG),weights=1/seBetaYG^2)$coef). I can guess the link between the two, though I have not checked the relationship. Still, running a toy simulation I noticed a substantially larger variance for the two-step approach as compared to the weighted linear regression. Please clarify, and update text and results if necessary.
2. Page 7. More details and some clarifications are welcome on the second paragraph. It starts by stating that “*The standard approach to estimating beta with* ***INDIVIDUAL LEVEL DATA*** *is Two Stage Least Squares…*” but a few lines below state that “*It is commonly used in two sample SUMMARY DATA MR out of necessity, but it is not typically used in the* ***ONE SAMPLE*** *setting...*” So is this the algorithm for the one sample individual level data, or the two sample summary statistics?

The text also says that “*TSLS firstly regress the exposure on all k genotypes under model (1)”* but model (1), which I understand is the generative model, also includes the confounding factor U. Same thing when referring to model (2). It would help to have the generative model and the model tested presented separately.

3. Page 8. There are several notions that should be clarified. (a) I’m not sure what the authors are referring to by “*X represents an inverted fork*”. (b) The “d-separation” term should be briefly defined. (c) The authors introduce the notion of “dilution”, which I assume is a bias of the (beta-beta*)_hat toward the null. Regarding the importance of this notion further in the manuscript, a formal definition is needed.
4. Page 8. It took me a bit of time and some toy simulations to apprehend the formulas for the proposed collider-bias correction (eq 4 to 7). Some illustration of the behavior of eq 6 would help. For example; in the absence of confounding effect (beta=beta*) and no direct G-Y association (alpha=0), then we get back the standard adjustment for the intermediate exposure and Exp[alpha_star]=0.

Also, equation (6) introduces alpha_0 which I understand is the intercept. Intuitively I guess this would equals Exp[alpha_j], which would equal to 0 under the InSIDE assumption?

5. Page 8, last paragraph. I am a little confuse by the statement: “*if the sample covariance Cov_hat(alpha_j, beta_XG) is zero (the InSIDE assumption [9]), then Cov(alpha*_j, beta_XGj) will also be zero.*” This seems to contradict equation (5) and (6), which explicitly show a linear relationship between *alpha*_j* and both *beta_XGj and beta_XGj_hat.* Please add a proof and provide the rationale in the main text.
6. Page 9, IVW implementation. “*setting the parameter alpha_0 to zero in equation (6)*”. Please expend the rationale --Cf comment 4
7. Page 9. More details and clarification are needed when defining the Cochran’s Q statistics. (a) please provide some rationale behind equation (9) and in the weighting scheme *w_j*. (b) I guessed the parameter in parenthesis –w_j(**0**), Q(**0**), Q(**1**), corresponds to the vector given in the denominator of *w_j*?
8. The authors introduce the MR-RAPS and MR-BESIDE only in the real data application. Having them in the simulation section would help understanding their performances in a controlled scenario.

Minor comments:

9. Page 5, typo: “*The* ***the*** *two-sample approach assumes…*”
10. Page 6, typo?: “*on either* ***or*** *men or women*”
11. Page 7, please refer to Figure 2 when describing the steps to obtain the IVW estimates
12. Page 8, section title typo? “*In****TR****oducing collider bias…*”
13. It looks like Ref 33 (Bowden et al) is now published in IJE
14. Page 9, please add a reference for using the F-stat to quantify the bias (e.g. PMID=21414999?)
15. Fig 3 & 4. Some of the curves (standard IVW, standard LAD, standard MR-Egger) are either missing or hidden behind existing one. Please find a way to show them –especially when referring to them in the text (e.g. page 13 “*Again, we see that standard and collider corrected MR-Egger method*”).
16. Page 12, last sentence. “*Collider-correction is shown to be far more efficient*” missing <in one sample> ?
