# Supplementary material for: Exploiting collider bias to apply two-sample summary data Mendelian randomization methods to one-sample individual level data
Source: PLoS Genet. 2021 Aug 9;17(8):e1009703. doi: 10.1371/journal.pgen.1009703 (PMC8376220; doi:10.1371/journal.pgen.1009703)
Supplement: Attachment — Submitted filename: ReviewerResponse_Final.docx [file pgen.1009703.s005.docx]

Review response

Dear Editor

Thank you for facilitating the review of our manuscript. We have attempted to address all of the reviewers’ comments in this revised version. Areas of the text which have been substantially changed are marked in blue.

Below we provide a point-by-point response to the reviewers’ queries. Our answers are shown in bold.

**Comments to the Authors:**

Reviewer #1: The manuscript presents an elegant use of the collider correction idea of Dudbridge et al., but instead of using it for SNP-effect correction, it is applied to remove bias in one sample causal effect estimation. While the MR field has focused tremendously on 2-sample MR methods due to data availability, one-sample MR is still only done by 2SLS. Since the bias correction is stated as a regression problem, analogously to most 2-sample MR methods, it can be combined with any such MR method – however estimating the causal effect, they now estimate the bias.

In all fairness, it has to be stated that the method still requires 2 samples, since one is needed to select the instruments and the method is applicable only when the instruments are known. The Winner’s curse bias of such instrument selection is not touched upon, which is acceptable, but needs to be stated upfront. The simulation results are convincing and the real data application shows a good example how such collider correction improves causal effect estimation. Below I list a few comments, some of which may improve the manuscript.

Major comments

How different is the method compared to using the Dudbridge et al [29] method to correct the G-Y summary statistics for collider bias and then use classical 2-sample MR methods for the corrected G-Y and G-X summary stats?

**Thank you. The approach outlined above would not estimate the causal effect (which our method does but the original Dudbridge method does not). In fact under the InSIDE assumption and balanced pleiotropy, the IVW estimate resulting from this strategy would be zero. This is because there is no collider bias under the standard MR set up, so the GY associations don’t need correcting.**

**We have added text to the end of the Introduction section to clarify the major differences between our approach and the approach of Dudbridge et al, which we agree weren’t clear enough**

The applicability of the method is rather limited: it requires a GWAS to be performed on a YadjX trait, hence its applicability to summary statistics is very low. This needs to be admitted in the Discussion.

**Whilst we agree with the reviewer’s point regarding the applicability to existing summary data sources, we see several additional applications for our approach**

**The first is when researchers have direct access to individual level patient data. This is likely to become much more common over time as further international biobanks start to release IPD. Extracting the summary statistics for our approach then enables the implementation of any two-sample method to the data. This is attractive because the methods to adjust for weak instrument bias and pleiotropy are far more advanced in the two sample setting than the one sample setting, as well as being more familiar to researchers.**

**The second is when authors do not have direct access to individual level data. In this case, we expect that researchers could request the summary statistics necessary to implement their approach directly from the data custodians, safe in the knowledge that they can reproduce the analysis in a fraction of the time, with obvious data security benefits.**

**We have added further text to the end of the introduction to make these points, as well as mentioning the applicability to YadjX summary data.**

The variance-bias tradeoff when adding the SIMEX correction for weak instrument bias could be explored further. Bias corrections are much less interesting in practice that lead to increased RMSE. Could the authors specify in what kind of settings would the RMSE of the SIMEX corrected estimate still decrease? E.g. in Fig 3 (bottom), how would the bias^2+SD^2 plots would look like for these methods?

**Thank-you for raising this important point. In our simulations we now report MSE as a function of sample size when implementing IVW, MR-Egger and LAD regression. We show that the simex-adjusted collider correction implementation of each method obtains minimum mean-squared error across all other analysis options, thus significantly strengthening the utility of the approach.**

It would be interesting to assess how much this approach may still suffer from Winner’s curse bias, which has been ignored as the (50) SNPs have been pre-selected. This is particularly important when the authors correct for the regression dilution bias of Eq (6): The mean F-statistic is much more biased in real data applications, when the SNPs are selected in the same data set: thus at each locus the top SNP is chosen, but for this the F-statistic is overestimated and hence the regression-dilution bias is underestimated. It would be key that the authors in their simulations use rather 50 loci (use realistic LD patterns at each locus) and choose the top hit SNPs as instruments, as people would do for real data. I strongly suspect that the SIMEX approach (or any other method to mitigate this bias) would perform worse. Also, loci that do not reach genome-wide (GW) significance are not used, hence not always all the 50 SNPs should be used as instruments. For real data examples, there are many hundreds of loci reach GW significance and such bias in the regression dilution estimation is far stronger. I’d invite the authors to include more loci and decide which ones to use as instruments that survive some threshold to reflect more realistic settings. I do not feel that extensive analysis of this phenomenon is needed, only some effort to show how serious this bias could be.

**We acknowledge the problem of winner’s curse in the paper. For example, in our applied analysis we present results where there is partial overlap in the data used to select instruments, and results where we have carefully selected the data to ensure there is no overlap. The former is affected by Winner’s curse bias but the latter is not. These results are compared and contrasted and we observe a bias towards the null, as theory suggests.**

**Our method allows any (future) approach that corrects winner’s curse in the 2-sample design to be used in the 1-sample design, but we feel that incorporating an additional winner’s curse correction is beyond the scope of this paper.**

In the real data application X is binary and logistic regression is used, while in the methods the models for X and Y assume linear models. How is this contradiction resolved?

**Thank you for pointing this out. We have added some text in Methods to explain how the causal estimands with a linear exposure change with a binary exposure. Specifically, a binary exposure does not pose any additional challenges to the implementation or interpretation of the causal effect. In both cases we are free to invoke Homogeneity as the fourth `point identifying’ assumption. In the Results section we again remind the reader that the causal effect relies on Homogeneity.**

**An alternative fourth IV assumption is `Monotonicity'. In this context it would state that, for each SNP used as an IV, there is no individual who would be exposed if they had a low genetic risk of exposure (i.e. no copies of the risk increasing allele) and who wouldn't be exposed if they had a high genetic risk of exposure (i.e. one or more copies of the exposure). If Monotonicity is satisfied, then the estimand can be interpreted as the causal effect in a subset of participants termed the Compliers. Compliers are individuals who would be exposed if they had a `high’ genetic risk of exposure (i.e. one or more of the risk increasing allele) and who wouldn't be exposed if they had a `low’ genetic risk of exposure (i.e. no copies of the risk increasing allele). For reasons of brevity we would prefer not to include a lengthy description of monotonicity in this paper.**

It was not clear in the real data application which of the methods listed in Table 1 were applied to the artificially induced Y~X+G based beta_GY summary statistics and whether they have directly applied classical MR methods to simple Y~G vs X~G types of summary stats (which has sample overlap bias) or to TSLS, which would be the standard choice? This is also not very clear in the simulations: when they say “standard IVW” is it IVW of the Y~G/X~G or Y~X+G/X~G estimates? I guess/hope it is the former one.

**We are sorry that this wasn’t clear. All methods with the prefix `Col-cor’ have been applied to the artificially induced Y~X+G based beta_GY** **statistics. Methods with the prefix `Standard’ have been applied to Y~G vs X~G summary statistics. We have changed the notation so that this is clearer.**

Minor comments
1. Black curves are not visible in Figs 3A, 4A/C. I know that it is overlapping other curves, but the reader cannot know which ones (maybe use dashed lines).

**We have replaced the solid lines with dashed lines as suggested so that the results of all methods are now clear**

2. In Eq (8), shouldn’t sigma^2 have a “hat” on it, since it is just an estimate for the variance of the estimator?

**Yes this is strictly true, although it has become convention to remove this from the notation in the MR literature (and the meta-analysis literature), as the uncertainty in this estimate is generally very small and assume it is `fixed and known’. We keep this convention but now remind the reader that this is an estimate.**

3. Why the standard error in Fig 3D collider corrected 1 sample IVW is increasing with the sample size? Would be informative to add the “collider uncorrected 2 sample” MR estimates to Fig 3B (bottom left), would it be the same as collider corrected 2 sample IVW?

**Thank you for raising this. To clarify, we are plotting the Monte-Carlo standard deviation of each estimate in Figure 3D. We suspect that the reason for this is due to a bias/variance trade-off. 1-sample collider-corrected IVW estimates have a smaller variation, but this is because they are more biased than 1-sample collider-corrected estimates with an additional weak instrument correction. We now make this point explicit in the text.**

**Regarding Figure 3B and Figure 3D: we are very sorry for the confusion. The two sample approaches should not have been given the pre-fix `Collider Correction’: That is, they show the results of a standard two sample analysis, where SNP-outcome associations are regressed on SNP-exposure associations using separate halves of the data. This has been corrected.**

4. “(a) The standard IVW estimate (black line); (b) the SIMEX adjusted standard IVW estimate (blue line); (c) the collider corrected IVW estimate (red line); (d) the collider Corrected IVW estimate with SIMEX correction (green line); and (e) the TSLS estimate (orange line). We see that methods (a), (c) and (e) give essentially the same answer and can therefore not be individually distinguished in the figure.” – I’m not sure I get it: collider correction does nothing to IVW? How is that possible?

**We have updated the figure to improve clarity. That is correct: collider correction does not change the point estimate, but it provides a means to easily adjust for weak instrument bias and pleiotropy within a single model. Therefore, whilst standard and collider corrected IVW estimates are the same on average, SIMEX adjusted estimates are substantially different.**

Reviewer #2: With pleasure I read this manuscript about using a correction for collider bias to apply two-sample summary data Mendelian randomization (MR) methods to one-sample individual level data. These MR methods are gaining a lot of traction and I believe the authors propose an idea that is likely to gain more traction, as the number of large datasets with individual data are becoming more and more available (think of UK Biobank, Biobank Japan and the Million Veterans program). However, I feel like in the current form the manuscript is somewhat puzzling. In a somewhat arbitrary order, I have listed my points below:

1. I feel like the method proposed by the authors is not compared to the right models. Currently, they only show how their method compares to a regular IVW (with/without SIMEX)/TSLS method without correcting for collider bias. However, I feel like I miss a lot of methods here that would be more interesting to compare the method to, such as Robinson’s 1988 partially linear model, limited information maximum likelihood, and semi parametric methods such as generalized methods of moments (GMM) and structural mean models (SMM).

**Thank you for your suggestion. Our aim in this paper is to showcase a simple trick that enables any contemporary two-sample summary data MR approach to be efficiently applied to single sample data. A key feature of these methods is their attempt to account for pleiotropy and weak instrument bias. Some methods contain in-built adjustments for weak instrument bias and others can be adjusted using general schemes such as SIMEX. Two methods with in-built adjustments are the IVW approach with exact weighting and MR-RAPS. Both approaches are equivalent to LIML applied to summary data**

**(see page 12 of** [**https://arxiv.org/pdf/1801.09652.pdf**](https://arxiv.org/pdf/1801.09652.pdf)**). We now make this point clear in the revised version. We now include an additional section in the manuscript to clarify this**

**A comparison of our approach to GMM and SMM approaches is an interesting avenue for further research but we believe it is outside the scope of the paper and the interest of the PLOS genetics readership.**

2. I think the current reporting of only the estimates is somewhat misleading, given that the standard deviations of the proposed method are much larger (as shown in the bottom right panel of Figure 3). I can imagine that in the current form, due to the large variance, just by chance this method can have a worse estimator compared to just doing a regular IVW. I think a measure that takes into account both bias and variance of the method such as mean squared prediction error (MSPE) (or some other metric as mean average prediction error (MAPE)) is more insightful.

**Thank you for raising this point (see also reviewer 1).** **In our simulations we now report MSE as a function of sample size when implementing IVW, MR-Egger and LAD regression. We show that the SIMEX-adjusted collider correction implementation of each method obtains minimum mean-squared error across all other analysis options, thus significantly strengthening the utility of the approach.**

3. I feel like the current empirical example is worrisome. The authors results are very prone to weak instrument bias (illustrated by the low F-statistics of 8.36 and 6.88 as shown in Table 1) and should be interpreted with a lot more caution.

**We are sorry for the confusion. The results are prone to weak instrument bias because of (a) limitations in the sample size and (b) our choice to use independent data to select SNPs as instruments and estimate their effect (thus avoiding winner’s curse). However, we believe the example precisely illustrates the major benefit of using collider correction – it enables winner’s curse, weak instrument bias and pleiotropy to be easily dealt with using standard two sample methods.**

4. Also, I feel like the example given where there is overlap between discovery sample and estimation sample is a bad example of how an MR study should be done (due to winner’s curse) and hence it would be a better showcase to only report the example where there is no overlap.

**Please see our earlier comment. In our applied example we include two sets of results: One which uses overlapping data to select the instruments and one which uses independent data (precisely to avoid Winner’s curse)**

5. The proposed method strongly hinges on the InSIDE assumption. I feel like a proper discussion of this assumption is missing.

**Thank you for raising this point. In the first submission of our manuscript we incorrectly stated that the collider correction method relies on the InSIDE assumption. This in fact is not the case. Collider correction enables the efficient application of any two-sample summary data MR method to single sample individual level data. If that method is MR-Egger (which relies on the InSIDE assumption), collider correction MR-Egger relies on the InSIDE assumption. If the method is LAD regression (a close analogue of weighted median which does not rely on InSIDE but rather a `majority valid’ assumption), then collider correction LAD regression relies on the same assumption.**

**We have significantly added to the description of the method to make this clear, and included an additional simulation study. We have also modified the simulation study investigating the performance of LAD regression so that InSIDE is violated too.**

6. A more thorough inspection of what SNPs are chosen as outliers (appendix C) would be interesting.

**Thank you.**

**We identified the outliers according to the Cochran’s Q statistics, which is often used to test for the presence of heterogeneity. If heterogeneity is detected, this provides evidence of horizontal pleiotropy. To take one step further, we identified the traits related to these 14 SNPs (i.e., 6 SNPs in the analyses of 23andMe significant SNPs only and 13 SNPs in the analyses of 23andMe + UK Biobank significant SNPs respectively), as well as, the corresponding genes where the 14 SNPs located from the GWAS Catalog (https://www.ebi.ac.uk/gwas/ ). Most of the SNPs are only associated with insomnia except rs10758593 (type 1 and type 2 diabetes), rs12917449 (type 2 diabetes), rs1861412 (BMI), and rs429358 (~70 traits), which are also associated with other traits. This provides convincing evidence of pleiotropy and again underlines the need to use pleiotropy robust methods. This discussion has been added to the paper and we include full details of this additional analysis in an Excel file as supplementary material.**

7. Elaborate more on the decision: `we propose to fit step 3 using Least-Absolute Deviation (LAD) regression instead of least squares.’ So that I understand why this decision is made.

**Thank you. We use this approach because the median of a set of numbers is known to minimise the absolute sum of errors (or the L1-norm). So weighted LAD regression, which uses L1 minimisation is a very close analogue of weighted median, but implemented within a formal regression model. This fact makes it possible to apply SIMEX, which works with a broad class of regression models. As far as we are aware, this is the first proposed method for obtaining a weak-instrument robust median-based estimate.**

Minor remarks:
8. There is inconsistency in the mathematic equations, they sometimes have a missing comma or a dot to end the sentence.

**Thank you for spotting these, we have gone over the manuscript thoroughly to tidy up the grammar.**

9. Figures do often not contain a 0 on the Y axis. This is misleading, especiall in Figure 4 right bottom panel, and Figure 3 top right panel.

**Thank you for spotting this. We have corrected these figures so that the Y-axis starts at zero.**

Reviewer #3: see attached file

This is a quite interesting work and I greatly appreciated the very nice review of the state-of-the-art in the background section. Altogether, the authors proposed a fairly intuitive solution to improve the MR framework, estimating the average collider bias across multiple weak instruments to correct the estimator of the causal effect of the exposure on the outcome. I am actually surprised this solution has not been proposed before. The manuscript is quite dense, and my comments are mostly about providing the reader more details about the methods.

1. When describing the standard IVW method, the authors describe a two-step approach using an inverse variance meta-analysis of the ratio BetaYG over BetaXG (background, Fig 2, and Methods). However, in the attached R code, it appears they are in practice using a weighted linear regression with intercept forced at 0 (e.g. summary(lm(BetaYG~-1+BetaXG),weights=1/seBetaYG^2)$coef). I can guess the link between the two, though I have not checked the relationship. Still, running a toy simulation I noticed a substantially larger variance for the two-step approach as compared to the weighted linear regression. Please clarify, and update text and results if necessary.

**Thank you for raising this. We agree that we were surprised when first learning of this fact. It is one of the nice features of summary data Mendelian randomization. The two approaches mentioned are statistically equivalent if, when performing the meta-analysis approach directly, one accounts for heterogeneity using a multiplicative random effects model. This is explained in in detail in Bowden et al (2017)** [**https://pubmed.ncbi.nlm.nih.gov/28114746/**](https://pubmed.ncbi.nlm.nih.gov/28114746/)**. Our paper now includes a more thorough description of this fact.**

2. Page 7. More details and some clarifications are welcome on the second paragraph. It starts by stating that “*The standard approach to estimating beta with* ***INDIVIDUAL LEVEL DATA*** *is Two Stage Least Squares…*” but a few lines below state that “*It is commonly used in two sample SUMMARY DATA MR out of necessity, but it is not typically used in the* ***ONE SAMPLE*** *setting...*” So is this the algorithm for the one sample individual level data, or the two sample summary statistics?

**The algorithm we propose either requires the individual level data in order to generate the requisite summary statistics to implement the method. However, these summary statistics could be supplied to the user without the individual level data. We now make this point clearer in the paper and earlier on.**

The text also says that “*TSLS firstly regress the exposure on all k genotypes under model (1)”* but model (1), which I understand is the generative model, also includes the confounding factor U. Same thing when referring to model (2). It would help to have the generative model and the model tested presented separately.

**We are sorry that the wording was confusing here. We meant to say that all data was generated under models (1)-(3), not that we analysed the data according to this model, and specifically that we adjusted for U. This has been corrected.**

3. Page 8. There are several notions that should be clarified. (a) I’m not sure what the authors are referring to by “*X represents an inverted fork*”. (b) The “d-separation” term should be briefly defined. (c) The authors introduce the notion of “dilution”, which I assume is a bias of the (beta-beta*)_hat toward the null. Regarding the importance of this notion further in the manuscript, a formal definition is needed.

**Thank you, for raising this. We have removed some of this unnecessarily technical jargon from the text. We have also clarified that dilution refers to bias towards the null.**

4. Page 8. It took me a bit of time and some toy simulations to apprehend the formulas for the proposed collider-bias correction (eq 4 to 7). Some illustration of the behavior of eq 6 would help. For example; in the absence of confounding effect (beta=beta*) and no direct G-Y association (alpha=0), then we get back the standard adjustment for the intermediate exposure and Exp[alpha_star]=0.

Also, equation (6) introduces alpha_0 which I understand is the intercept. Intuitively I guess this would equals Exp[alpha_j], which would equal to 0 under the InSIDE assumption?

**Thank for raising these points. We have included an additional paragraph in the discussion to make these excellent points clear**

**“A nice feature of our approach is that the Collider-Correction term will be large (and therefore the Collider-Corrected estimate will be clearly distinct from the observational association) precisely when there is strong confounding. Conversely, when there is weak confounding, or the confounding has been sufficiently adjusted for, will be zero and Collider-Correction estimate will equal the observational association. In this case, the observational association then becomes a consistent and likely very efficient estimate of the true causal effect. Collider-Correction therefore naturally promotes the triangulation and synthesis of observational and MR estimates, which can estimate the true causal effect with distinct but complementary assumptions”**

**We have tried to clarify more clearly in the text the role of \alpha_0. This is generally set to zero for most MR methods, but is estimated within MR-Egger regression as a means of bias adjustment.**

5. Page 8, last paragraph. I am a little confuse by the statement: “*if the sample covariance Cov_hat(alpha_j, beta_XG) is zero (the InSIDE assumption [9]), then Cov(alpha*_j, beta_XGj) will also be zero.*” This seems to contradict equation (5) and (6), which explicitly show a linear relationship between *alpha*_j* and both *beta_XGj and beta_XGj_hat.* Please add a proof and provide the rationale in the main text.

**Thank you for raising this. This was indeed an error. We have made extensive changes to the methods section of the paper and included an additional simulation study to clarify this point.**

6. Page 9, IVW implementation. “*setting the parameter alpha_0 to zero in equation (6)*”. Please expend the rationale --Cf comment 4

**We set the intercept to zero to perform IVW regression, because the IVW estimate assumes balanced pleiotropy under the InSIDE assumption. We allow the intercept to be estimated when peforming MR-Egger, because MR-Egger adjusts for non-zero mean pleiotropy under the InSIDE assumption. We again set it to zero when performing LAD regression because this method assumes that the majority of SNPs are valid, and hence the intercept is zero when based on the majority of SNPS**

7. Page 9. More details and clarification are needed when defining the Cochran’s Q statistics. (a) please provide some rationale behind equation (9) and in the weighting scheme *w_j*. (b) I guessed the parameter in parenthesis –w_j(**0**), Q(**0**), Q(**1**), corresponds to the vector given in the denominator of *w_j*?

**Thank you for raising this. We have made extensive changes to the methods section of the paper. This includes removing a description of the extended Q statistic formulae and replacing it with an alternative regression-model-based alternative.**

8. The authors introduce the MR-RAPS and MR-BESIDE only in the real data application. Having them in the simulation section would help understanding their performances in a controlled scenario.

**The central argument of our paper is that collider correction can be applied to any two-sample summary data method, with the assumptions, strengths and limitations of the method transferring across to collider correction. MR-BESIDE is a computationally intensive MCMC-based Bayesian approach that takes several minutes to run, and it is infeasible to implement the method in a simulation study. Our paper now includes a more thorough description of the MR-RAPS approach, its connection to LIML (see earlier reviewers comment) , and simulation results that compare it to both the Collider-Corrected IVW and LAD regression approaches. We have also removed the MR-BESIDE approach from the paper for clarity.**

Minor comments:

9. Page 5, typo: “*The* ***the*** *two-sample approach assumes…*” **Corrected**
10. Page 6, typo?: “*on either* ***or*** *men or women*” **Corrected**
11. Page 7, please refer to Figure 2 when describing the steps to obtain the IVW estimates **Corrected**
12. Page 8, section title typo? “*In****TR****oducing collider bias…*” **Corrected**
13. It looks like Ref 33 (Bowden et al) is now published in IJE **Corrected**
14. Page 9, please add a reference for using the F-stat to quantify the bias (e.g. PMID=21414999?) **Corrected**
15. Fig 3 & 4. Some of the curves (standard IVW, standard LAD, standard MR-Egger) are either missing or hidden behind existing one. Please find a way to show them –especially when referring to them in the text (e.g. page 13 “*Again, we see that standard and collider corrected MR-Egger method*”). **Corrected**
16. Page 12, last sentence. “*Collider-correction is shown to be far more efficient*” missing <in one sample> ? **Corrected**
