# Supplementary material for: Exploiting collider bias to apply two-sample summary data Mendelian randomization methods to one-sample individual level data
Source: PLoS Genet. 2021 Aug 9;17(8):e1009703. doi: 10.1371/journal.pgen.1009703 (PMC8376220; doi:10.1371/journal.pgen.1009703)
Supplement: Attachment — Submitted filename: ReviewerResponse_Round2.docx [file pgen.1009703.s006.docx]

Reviewer Response

We thank the reviewers and editors for re-reviewing our manuscript. Please find below a point-by-point response to the reviewers’ comments.

Reviewer #1:

*I thank the authors for having addressed all my concerns. I have only a minor point left to be clarified: when the outcome (Y) is binary and a YadjX~G is done via logistic regression. I do not see how the derivation starting off from Eq (3) could be adapted, since there is some (non-linear) link function needs to be applied to the liability, Eq 5 would no longer hold in its form which assumes simple linear relationship between (X, G) and Y. I do not see how monotonicity can resolve this issue.*

Thank you for raising this important point. We have inserted the following paragraph in the discussion of the paper (shown in red) to acknowledge this fact, include two further references and offer three solutions to the reader.

“Often in MR analyses the outcome of interest is binary and a logistic regression model is used in place of the linear model to estimate the causal effect on the odds ratio scale. In this case, the interpretation of causal estimates from a resulting Collider-Correction analysis will be more nuanced for the following reason. Even if we replaced the assumed linear outcome model in equation (2) with a logistic model, so that \beta reflected the true causal log-odds ratio for a unit increase in the exposure experienced by each individual, the causal effect estimate (which is a population average) will be diluted by a factor that is proportional to the variance of the residual error in the model not explained by the genetically predicted exposure. This is due to the fact that the odds ratio is a non-collapsible measure (Vansteelandt et al, 2011). Although this dilution is a very general phenomenon that affects all logistic regression based analysis, three obvious options exist to the applied researcher if implementing Collider-Correction in the binary outcome case. The first would be to simply accept the interpretation of the causal estimate as a population average effect. The second would be to attempt to better approximate the individual causal effect by additionally adjusting for the first stage residual, (that is the observed exposure minus its genetically predicted value) in the second stage logistic model. This is referred to as the Control Function or adjusted IV approach (Palmer et al, 2008). The third option would be to estimate the causal effect on a risk difference scale. Since the risk difference is a collapsible measure, individual and population average effects are the same. Risk difference estimates can be estimated either by fitting a linear probability model or by extracting the risk difference contrast from the logistic model. This latter approach can be implemented using the margins() package in R . A thorough investigation of the performance of Collider-Correction in the binary outcome setting is an interesting avenue for future research.”

Reviewer #2:

*I want to congratulate the authors on improving the manuscript.
There are still some remarks that I would like the authors to clarify:

1. I want to stress that the authors need to be clear if they do or do not require the Inside assumption. Currently, they state in the response to reviewers they do not need this, but in the main manuscript on page 13 they still seem to use it ("under the assumption that the mean pleiotropic effect is zero and the InSIDE assumption is satisfed, the residual error independence property of Collider-Correction
will mean that ..."). I think this is a very important point to make, what assumptions does the method rely on.*

We are sorry for the continued confusion. In our revised manuscript we tried to make the following points clear:

(a) Any specific two sample summary data MR method will require an assumption to be made about the nature of pleiotropy for it to unbiasedly estimate the causal effect. For example, the IVW method requires that if pleiotropy is present it satisfies the InSIDE assumption and is balanced. Or alternatively, the weighted median approach assumes that the majority of the weight in the analysis stems from non-pleiotropic SNPs, but does not require InSIDE.
(b) Collider correction is a method for applying any two sample summary data MR method to one sample data, which is valid whenever the corresponding assumptions regarding pleiotropy made by the two sample approach are satisfied.
(c) Collider Correction does not impose any additional assumptions about the nature of the pleiotropy , over and above that made by the two sample method

It was for this reason we included an additional simulation (and Figure 3) which showed that the residual error independence property was preserved even when we generated data that violated InSIDE assumption. Following this we included the following paragraph

“In the toy example above we purposefully generated the data so that the InSIDE assumption was violated across the entire set of SNPs to demonstrate that residual error independence does not rely on InSIDE. However, the success of any subsequently applied Collider-Corrected two sample approach in consistently estimating the causal effect β (i.e. so that it is asymptotically unbiased) will of course depend on the pleiotropy identifying assumption being met, just as if it were being applied in a standard two-sample setting”

So the answer is `no’, Collider-Correction does not intrinsically rely on the InSIDE assumption.

2. *I would like to know under what scenario's with pleiotropy the method will be worse compared to a (standard) IVW approach (please relate this to equation (10)).*

The Collider-Corrected IVW approach will in general be near-identical to the one sample IVW approach with strong instruments. Major differences are revealed in the presence of weak instruments and a weak instrument correction such as SIMEX is enacted. In this case, we see that the collider corrected SIMEX adjusted IVW estimate is minimally biased compared to the SIMEX adjusted IVW approach. Put simply, weak instrument bias corrections do not work with one sample data unless Collider Correction is first enacted. These points are shown in Figure 4 and the related discussion.

3. *How prone is the method to weak-instrument bias? There are some hints to this throughout the manuscript, but it is unclear to me if the method is more or less susceptible to this.*

The Collider Correction algorithm is no more or less prone to weak instrument bias than the underlying two-sample approach it uses. However, Collider-Correction makes it simple and straightforward to adjust for weak instrument bias if it is present. See point 2.

*Minor remark: figure reference is missing on page 13: " Figure (top-left) shows, for a range of sample sizes the average value across 1000 independent data sets of ... "*

Thank you for spotting this, this has been corrected.
