# Supplementary material for: Exploiting collider bias to apply two-sample summary data Mendelian randomization methods to one-sample individual level data
Source: PLoS Genet. 2021 Aug 9;17(8):e1009703. doi: 10.1371/journal.pgen.1009703 (PMC8376220; doi:10.1371/journal.pgen.1009703)
Supplement: Peer review history

# Peer review: Decision Letter 0

**DOI:** 10.1371/journal.pgen.1009703.r001

**Authors:** David Balding, Heather J Cordell

23 Mar 2021

Dear Dr Bowden,

Thank you very much for submitting your Methods entitled 'Exploiting collider bias to apply two-sample summary data Mendelian randomization methods to one-sample individual level data' to PLOS Genetics.  We apologise for the slow response, largely due to waiting for a late review but we think it was helpful and worth waiting for.

The reviewers appreciated the attention to an important problem, but raised some substantial concerns about the current manuscript. Based on the reviews, we will not be able to accept this version of the manuscript, but we would be willing to review a much-revised version. We cannot, of course, promise publication at that time.

Should you decide to revise the manuscript for further consideration here, your revisions should address the specific points made by each reviewer. We will also require a detailed list of your responses to the review comments and a description of the changes you have made in the manuscript.

If you decide to revise the manuscript for further consideration at PLOS Genetics, please aim to resubmit within the next 60 days, unless it will take extra time to address the concerns of the reviewers, in which case we would appreciate an expected resubmission date by email to plosgenetics@plos.org.

If present, accompanying reviewer attachments are included with this email; please notify the journal office if any appear to be missing. They will also be available for download from the link below. You can use this link to log into the system when you are ready to submit a revised version, having first consulted our Submission Checklist.

To enhance the reproducibility of your results, we recommend that you deposit your laboratory protocols in protocols.io, where a protocol can be assigned its own identifier (DOI) such that it can be cited independently in the future. Additionally, PLOS ONE offers an option to publish peer-reviewed clinical study protocols. Read more information on sharing protocols at https://plos.org/protocols?utm_medium=editorial-email&utm_source=authorletters&utm_campaign=protocols

Please be aware that our data availability policy requires that all numerical data underlying graphs or summary statistics are included with the submission, and you will need to provide this upon resubmission if not already present. In addition, we do not permit the inclusion of phrases such as "data not shown" or "unpublished results" in manuscripts. All points should be backed up by data provided with the submission.

While revising your submission, please upload your figure files to the Preflight Analysis and Conversion Engine (PACE) digital diagnostic tool.  PACE helps ensure that figures meet PLOS requirements. To use PACE, you must first register as a user. Then, login and navigate to the UPLOAD tab, where you will find detailed instructions on how to use the tool. If you encounter any issues or have any questions when using PACE, please email us at figures@plos.org.

PLOS has incorporated Similarity Check, powered by iThenticate, into its journal-wide submission system in order to screen submitted content for originality before publication. Each PLOS journal undertakes screening on a proportion of submitted articles. You will be contacted if needed following the screening process.

To resubmit, use the link below and 'Revise Submission' in the 'Submissions Needing Revision' folder.

[LINK]

We are sorry that we cannot be more positive about your manuscript at this stage. Please do not hesitate to contact us if you have any concerns or questions.

Yours sincerely,

Heather J Cordell

Associate Editor

PLOS Genetics

David Balding

Section Editor: Methods

PLOS Genetics

Reviewer's Responses to Questions

**Comments to the Authors:**

Reviewer #1: The manuscript presents an elegant use of the collider correction idea of Dudbridge et al., but instead of using it for SNP-effect correction, it is applied to remove bias in one sample causal effect estimation. While the MR field has focused tremendously on 2-sample MR methods due to data availability, one-sample MR is still only done by 2SLS. Since the bias correction is stated as a regression problem, analogously to most 2-sample MR methods, it can be combined with any such MR method – however estimating the causal effect, they now estimate the bias.

In all fairness, it has to be stated that the method still requires 2 samples, since one is needed to select the instruments and the method is applicable only when the instruments are known. The Winner’s curse bias of such instrument selection is not touched upon, which is acceptable, but needs to be stated upfront. The simulation results are convincing and the real data application shows a good example how such collider correction improves causal effect estimation. Below I list a few comments, some of which may improve the manuscript.

Major comments

How different is the method compared to using the Dudbridge et al [29] method to correct the G-Y summary statistics for collider bias and then use classical 2-sample MR methods for the corrected G-Y and G-X summary stats?

The applicability of the method is rather limited: it requires a GWAS to be performed on a YadjX trait, hence its applicability to summary statistics is very low. This needs to be admitted in the Discussion.

The variance-bias tradeoff when adding the SIMEX correction for weak instrument bias could be explored further. Bias corrections are much less interesting in practice that lead to increased RMSE. Could the authors specify in what kind of settings would the RMSE of the SIMEX corrected estimate still decrease? E.g. in Fig 3 (bottom), how would the bias^2+SD^2 plots would look like for these methods?

It would be interesting to assess how much this approach may still suffer from Winner’s curse bias, which has been ignored as the (50) SNPs have been pre-selected. This is particularly important when the authors correct for the regression dilution bias of Eq (6): The mean F-statistic is much more biased in real data applications, when the SNPs are selected in the same data set: thus at each locus the top SNP is chosen, but for this the F-statistic is overestimated and hence the regression-dilution bias is underestimated. It would be key that the authors in their simulations use rather 50 loci (use realistic LD patterns at each locus) and choose the top hit SNPs as instruments, as people would do for real data. I strongly suspect that the SIMEX approach (or any other method to mitigate this bias) would perform worse. Also, loci that do not reach genome-wide (GW) significance are not used, hence not always all the 50 SNPs should be used as instruments. For real data examples, there are many hundreds of loci reach GW significance and such bias in the regression dilution estimation is far stronger. I’d invite the authors to include more loci and decide which ones to use as instruments that survive some threshold to reflect more realistic settings. I do not feel that extensive analysis of this phenomenon is needed, only some effort to show how serious this bias could be.

In the real data application X is binary and logistic regression is used, while in the methods the models for X and Y assume linear models. How is this contradiction resolved?

It was not clear in the real data application which of the methods listed in Table 1 were applied to the artificially induced Y~X+G based beta_GY summary statistics and whether they have directly applied classical MR methods to simple Y~G vs X~G types of summary stats (which has sample overlap bias) or to TSLS, which would be the standard choice? This is also not very clear in the simulations: when they say “standard IVW” is it IVW of the Y~G/X~G or Y~X+G/X~G estimates? I guess/hope it is the former one.

Minor comments

1. Black curves are not visible in Figs 3A, 4A/C. I know that it is overlapping other curves, but the reader cannot know which ones (maybe use dashed lines).

2. In Eq (8), shouldn’t sigma^2 have a “hat” on it, since it is just an estimate for the variance of the estimator?

3. Why the standard error in Fig 3D collider corrected 1 sample IVW is increasing with the sample size? Would be informative to add the “collider uncorrected 2 sample” MR estimates to Fig 3B (bottom left), would it be the same as collider corrected 2 sample IVW?

4. “(a) The standard IVW estimate (black line); (b) the SIMEX adjusted standard IVW estimate (blue line); (c) the collider corrected IVW estimate (red line); (d) the collider Corrected IVW estimate with SIMEX correction (green line); and (e) the TSLS estimate (orange line). We see that methods (a), (c) and (e) give essentially the same answer and can therefore not be individually distinguished in the figure.” – I’m not sure I get it: collider correction does nothing to IVW? How is that possible?

Reviewer #2: With pleasure I read this manuscript about using a correction for collider bias to apply two-sample summary data Mendelian randomization (MR) methods to one-sample individual level data. These MR methods are gaining a lot of traction and I believe the authors propose an idea that is likely to gain more traction, as the number of large datasets with individual data are becoming more and more available (think of UK Biobank, Biobank Japan and the Million Veterans program).

However, I feel like in the current form the manuscript is somewhat puzzling. In a somewhat arbitrary order, I have listed my points below:

1. I feel like the method proposed by the authors is not compared to the right models. Currently, they only show how their method compares to a regular IVW (with/without SIMEX)/TSLS method without correcting for collider bias. However, I feel like I miss a lot of methods here that would be more interesting to compare the method to, such as Robinson’s 1988 partially linear model, limited information maximum likelihood, and semi parametric methods such as generalized methods of moments (GMM) and structural mean models (SMM).

2. I think the current reporting of only the estimates is somewhat misleading, given that the standard deviations of the proposed method are much larger (as shown in the bottom right panel of Figure 3). I can imagine that in the current form, due to the large variance, just by chance this method can have a worse estimator compared to just doing a regular IVW. I think a measure that takes into account both bias and variance of the method such as mean squared prediction error (MSPE) (or some other metric as mean average prediction error (MAPE)) is more insightful.

3. I feel like the current empirical example is worrisome. The authors results are very prone to weak instrument bias (illustrated by the low F-statistics of 8.36 and 6.88 as shown in Table 1) and should be interpreted with a lot more caution.

4. Also, I feel like the example given where there is overlap between discovery sample and estimation sample is a bad example of how an MR study should be done (due to winner’s curse) and hence it would be a better showcase to only report the example where there is no overlap.

5. The proposed method strongly hinges on the InSIDE assumption. I feel like a proper discussion of this assumption is missing.

6. A more thorough inspection of what SNPs are chosen as outliers (appendix C) would be interesting.

7. Elaborate more on the decision: `we propose to fit step 3 using Least-Absolute Deviation (LAD) regression instead of least squares.’ So that I understand why this decision is made.

Minor remarks:

8. There is inconsistency in the mathematic equations, they sometimes have a missing comma or a dot to end the sentence.

9. Figures do often not contain a 0 on the Y axis. This is misleading, especiall in Figure 4 right bottom panel, and Figure 3 top right panel.

Reviewer #3: see attached file

**********

**Have all data underlying the figures and results presented in the manuscript been provided?**

Large-scale datasets should be made available via a public repository as described in the *PLOS Genetics* data availability policy, and numerical data that underlies graphs or summary statistics should be provided in spreadsheet form as supporting information.

Reviewer #1: Yes

Reviewer #2: Yes

Reviewer #3: Yes

**********

PLOS authors have the option to publish the peer review history of their article (what does this mean?). If published, this will include your full peer review and any attached files.

If you choose “no”, your identity will remain anonymous but your review may still be made public.

**Do you want your identity to be public for this peer review?** For information about this choice, including consent withdrawal, please see our Privacy Policy.

Reviewer #1: No

Reviewer #2: No

Reviewer #3: No

Attachment Submitted filename: review.docx Click here for additional data file.

# Author comment: Author response to Decision Letter 0

**DOI:** 10.1371/journal.pgen.1009703.r002

26 May 2021

Attachment Submitted filename: ReviewerResponse_Final.docx Click here for additional data file.

# Peer review: Decision Letter 1

**DOI:** 10.1371/journal.pgen.1009703.r003

**Authors:** David Balding, Heather J Cordell

20 Jun 2021

Dear Dr Bowden,

Thank you very much for submitting your Methods entitled 'Exploiting collider bias to apply two-sample summary data Mendelian randomization methods to one-sample individual level data' to PLOS Genetics.

The manuscript was fully evaluated at the editorial level and by independent peer reviewers. The reviewers appreciated the improvements made in your revised manuscript but identified some remaining concerns.

We therefore ask you to revise the manuscript in the light of the reviewer recommendations. You should address the specific points made by each reviewer, either in the manuscript or through an explanation in your covering letter.  The editors have some concern that in trying to respond to previous reviewer comments, the manuscript has lost some readability and so we encourage you to review the manuscript for opportunities to improve clarity.  We note again that it's not necessary to make a change suggested by a reviewer if you can give a good explanation why not.  The manuscript is also rather long and with many figures, please consider whether some material can be moved to supplementary information.

In addition we ask that you:

1) Provide a detailed list of your responses to the review comments and a description of the changes you have made in the manuscript.

2) Upload a Striking Image with a corresponding caption to accompany your manuscript if one is available (either a new image or an existing one from within your manuscript). If this image is judged to be suitable, it may be featured on our website. Images should ideally be high resolution, eye-catching, single panel square images. For examples, please browse our archive. If your image is from someone other than yourself, please ensure that the artist has read and agreed to the terms and conditions of the Creative Commons Attribution License. Note: we cannot publish copyrighted images.

We hope to receive your revised manuscript within the next 30 days. If you anticipate any delay in its return, we would ask you to let us know the expected resubmission date by email to plosgenetics@plos.org.

If present, accompanying reviewer attachments should be included with this email; please notify the journal office if any appear to be missing. They will also be available for download from the link below. You can use this link to log into the system when you are ready to submit a revised version, having first consulted our Submission Checklist.

While revising your submission, please upload your figure files to the Preflight Analysis and Conversion Engine (PACE) digital diagnostic tool. PACE helps ensure that figures meet PLOS requirements. To use PACE, you must first register as a user. Then, login and navigate to the UPLOAD tab, where you will find detailed instructions on how to use the tool. If you encounter any issues or have any questions when using PACE, please email us at figures@plos.org.

Please be aware that our data availability policy requires that all numerical data underlying graphs or summary statistics are included with the submission, and you will need to provide this upon resubmission if not already present. In addition, we do not permit the inclusion of phrases such as "data not shown" or "unpublished results" in manuscripts. All points should be backed up by data provided with the submission.

To enhance the reproducibility of your results, we recommend that you deposit your laboratory protocols in protocols.io, where a protocol can be assigned its own identifier (DOI) such that it can be cited independently in the future. Additionally, PLOS ONE offers an option to publish peer-reviewed clinical study protocols. Read more information on sharing protocols at https://plos.org/protocols?utm_medium=editorial-email&utm_source=authorletters&utm_campaign=protocols

Please review your reference list to ensure that it is complete and correct. If you have cited papers that have been retracted, please include the rationale for doing so in the manuscript text, or remove these references and replace them with relevant current references. Any changes to the reference list should be mentioned in the rebuttal letter that accompanies your revised manuscript. If you need to cite a retracted article, indicate the article’s retracted status in the References list and also include a citation and full reference for the retraction notice.

PLOS has incorporated Similarity Check, powered by iThenticate, into its journal-wide submission system in order to screen submitted content for originality before publication. Each PLOS journal undertakes screening on a proportion of submitted articles. You will be contacted if needed following the screening process.

To resubmit, you will need to go to the link below and 'Revise Submission' in the 'Submissions Needing Revision' folder.

[LINK]

Please let us know if you have any questions while making these revisions.

Yours sincerely,

Heather J Cordell

Associate Editor

PLOS Genetics

David Balding

Section Editor: Methods

PLOS Genetics

Reviewer's Responses to Questions

**Comments to the Authors:**

Reviewer #1: I thank the authors for having addressed all my concerns. I have only a minor point left to be clarified: when the outcome (Y) is binary and a YadjX~G is done via logistic regression. I do not see how the derivation starting off from Eq (3) could be adapted, since there is some (non-linear) link function needs to be applied to the liability, Eq 5 would no longer hold in its form which assumes simple linear relationship between (X, G) and Y. I do not see how monotonicity can resolve this issue.

Reviewer #2: I want to congratulate the authors on improving the manuscript.

There are still some remarks that I would like the authors to clarify:

1. I want to stress that the authors need to be clear if they do or do not require the Inside assumption. Currently, they state in the response to reviewers they do not need this, but in the main manuscript on page 13 they still seem to use it ("under the assumption that the mean pleiotropic effect is zero and the InSIDE assumption is satisfed, the residual error independence property of Collider-Correction

will mean that ..."). I think this is a very important point to make, what assumptions does the method rely on.

2. I would like to know under what scenario's with pleiotropy the method will be worse compared to a (standard) IVW approach (please relate this to equation (10)).

3. How prone is the method to weak-instrument bias? There are some hints to this throughout the manuscript, but it is unclear to me if the method is more or less susceptible to this.

Minor remark: figure reference is missing on page 13: " Figure (top-left) shows, for a range of sample sizes the average value across 1000 independent data sets of ... "

Reviewer #3: I thank the authors for their extensive response to my questions. I do not have further comments.

**********

**Have all data underlying the figures and results presented in the manuscript been provided?**

Large-scale datasets should be made available via a public repository as described in the *PLOS Genetics* data availability policy, and numerical data that underlies graphs or summary statistics should be provided in spreadsheet form as supporting information.

Reviewer #1: Yes

Reviewer #2: Yes

Reviewer #3: Yes

**********

PLOS authors have the option to publish the peer review history of their article (what does this mean?). If published, this will include your full peer review and any attached files.

If you choose “no”, your identity will remain anonymous but your review may still be made public.

**Do you want your identity to be public for this peer review?** For information about this choice, including consent withdrawal, please see our Privacy Policy.

Reviewer #1: No

Reviewer #2: No

Reviewer #3: No

# Author comment: Author response to Decision Letter 1

**DOI:** 10.1371/journal.pgen.1009703.r004

29 Jun 2021

Attachment Submitted filename: ReviewerResponse_Round2.docx Click here for additional data file.

# Peer review: Decision Letter 2

**DOI:** 10.1371/journal.pgen.1009703.r005

**Authors:** David Balding, Heather J Cordell

8 Jul 2021

Dear Dr Bowden,

We are pleased to inform you that your manuscript entitled "Exploiting collider bias to apply two-sample summary data Mendelian randomization methods to one-sample individual level data" has been editorially accepted for publication in PLOS Genetics. Congratulations!

Before your submission can be formally accepted and sent to production you will need to complete our formatting changes, which you will receive in a follow up email. Please be aware that it may take several days for you to receive this email; during this time no action is required by you. Please note: the accept date on your published article will reflect the date of this provisional acceptance, but your manuscript will not be scheduled for publication until the required changes have been made.

Once your paper is formally accepted, an uncorrected proof of your manuscript will be published online ahead of the final version, unless you’ve already opted out via the online submission form. If, for any reason, you do not want an earlier version of your manuscript published online or are unsure if you have already indicated as such, please let the journal staff know immediately at plosgenetics@plos.org.

In the meantime, please log into Editorial Manager at https://www.editorialmanager.com/pgenetics/, click the "Update My Information" link at the top of the page, and update your user information to ensure an efficient production and billing process. Note that PLOS requires an ORCID iD for all corresponding authors. Therefore, please ensure that you have an ORCID iD and that it is validated in Editorial Manager. To do this, go to ‘Update my Information’ (in the upper left-hand corner of the main menu), and click on the Fetch/Validate link next to the ORCID field.  This will take you to the ORCID site and allow you to create a new iD or authenticate a pre-existing iD in Editorial Manager.

If you have a press-related query, or would like to know about making your underlying data available (as you will be aware, this is required for publication), please see the end of this email. If your institution or institutions have a press office, please notify them about your upcoming article at this point, to enable them to help maximise its impact. Inform journal staff as soon as possible if you are preparing a press release for your article and need a publication date.

Thank you again for supporting open-access publishing; we are looking forward to publishing your work in PLOS Genetics!

Yours sincerely,

Heather J Cordell

Associate Editor

PLOS Genetics

David Balding

Section Editor: Methods

PLOS Genetics

www.plosgenetics.org

Twitter: @PLOSGenetics

----------------------------------------------------

Comments from the reviewers (if applicable):

Reviewer's Responses to Questions

**Comments to the Authors:**

Reviewer #1: I'd like to thank the authors for addressing my remaining point.

**********

**Have all data underlying the figures and results presented in the manuscript been provided?**

Large-scale datasets should be made available via a public repository as described in the *PLOS Genetics* data availability policy, and numerical data that underlies graphs or summary statistics should be provided in spreadsheet form as supporting information.

Reviewer #1: Yes

**********

PLOS authors have the option to publish the peer review history of their article (what does this mean?). If published, this will include your full peer review and any attached files.

If you choose “no”, your identity will remain anonymous but your review may still be made public.

**Do you want your identity to be public for this peer review?** For information about this choice, including consent withdrawal, please see our Privacy Policy.

Reviewer #1: No

----------------------------------------------------

**Data Deposition**

If you have submitted a Research Article or Front Matter that has associated data that are not suitable for deposition in a subject-specific public repository (such as GenBank or ArrayExpress), one way to make that data available is to deposit it in the Dryad Digital Repository. As you may recall, we ask all authors to agree to make data available; this is one way to achieve that. A full list of recommended repositories can be found on our website.

The following link will take you to the Dryad record for your article, so you won't have to re‐enter its bibliographic information, and can upload your files directly: 

http://datadryad.org/submit?journalID=pgenetics&manu=PGENETICS-D-20-01817R2

More information about depositing data in Dryad is available at http://www.datadryad.org/depositing. If you experience any difficulties in submitting your data, please contact help@datadryad.org for support.

Additionally, please be aware that our data availability policy requires that all numerical data underlying display items are included with the submission, and you will need to provide this before we can formally accept your manuscript, if not already present.

----------------------------------------------------

**Press Queries**

If you or your institution will be preparing press materials for this manuscript, or if you need to know your paper's publication date for media purposes, please inform the journal staff as soon as possible so that your submission can be scheduled accordingly. Your manuscript will remain under a strict press embargo until the publication date and time. This means an early version of your manuscript will not be published ahead of your final version. PLOS Genetics may also choose to issue a press release for your article. If there's anything the journal should know or you'd like more information, please get in touch via plosgenetics@plos.org.

# Editor report: Acceptance letter

**DOI:** 10.1371/journal.pgen.1009703.r006

**Authors:** David Balding, Heather J Cordell

4 Aug 2021

PGENETICS-D-20-01817R2 

Exploiting collider bias to apply two-sample summary data Mendelian randomization methods to one-sample individual level data 

Dear Dr Bowden, 

We are pleased to inform you that your manuscript entitled "Exploiting collider bias to apply two-sample summary data Mendelian randomization methods to one-sample individual level data" has been formally accepted for publication in PLOS Genetics! Your manuscript is now with our production department and you will be notified of the publication date in due course.

The corresponding author will soon be receiving a typeset proof for review, to ensure errors have not been introduced during production. Please review the PDF proof of your manuscript carefully, as this is the last chance to correct any errors. Please note that major changes, or those which affect the scientific understanding of the work, will likely cause delays to the publication date of your manuscript. 

Soon after your final files are uploaded, unless you have opted out or your manuscript is a front-matter piece, the early version of your manuscript will be published online. The date of the early version will be your article's publication date. The final article will be published to the same URL, and all versions of the paper will be accessible to readers.

Thank you again for supporting PLOS Genetics and open-access publishing. We are looking forward to publishing your work! 

With kind regards,

Melanie Wincott

PLOS Genetics

On behalf of:

The PLOS Genetics Team

Carlyle House, Carlyle Road, Cambridge CB4 3DN | United Kingdom

plosgenetics@plos.org | +44 (0) 1223-442823

plosgenetics.org | Twitter: @PLOSGenetics
